# Mental health crises among youth in the context of migration – a cross-sectional study of psychiatric emergencies

**DOI:** 10.1186/s12889-026-26964-7

**Published:** 2026-03-19

**Authors:** Michelle Pantis, Arabella Bergmann, Elina Mizgir, Daniela Hagmann, Priska Schneider, Gottfried Maria Barth, Tobias J. Renner, Katharina Allgaier

**Affiliations:** 1 Department of Child and Adolescent Psychiatry,Psychosomatics and Psychotherapy, University Hospital of Psychiatry and Psychotherapy Tübingen, Tübingen, Germany; 2 German Center for Mental Health (DZPG) partner site Tübingen, Tübingen, Germany

**Keywords:** Children and adolescents, Emergencies, Migration, Mental health crises, Predictors

## Abstract

**Background:**

Adolescents face various challenges that can negatively affect their mental health. Unmanaged stress may lead to mental health crises. Adolescents with a migration background may face additional challenges, making them especially vulnerable and at increased risk for mental health disorders. As migrant adolescents represent a considerable proportion of the adolescent population worldwide, including in Germany, it is crucial to ensure accessible and appropriate mental health care in order to prevent mental health crises in adolescent migrants. It is thus essential to identify specific stressors among migrant adolescents. However, the few current findings on mental health crises in this group remain heterogeneous, highlighting the need for further research. We analyzed the sample characteristics of migrant youth presenting as emergencies at the Child- and Adolescent Psychiatry (CAP) in Tübingen, Germany. We additionally applied exploratory analyses with migration background as a correlate of psychosocial burden to determine which stress factors were particularly likely to be associated with mental health crises in migrant youth.

**Methods:**

We used cross-sectional data from patients (mean age = 14.7 years; 76% female) with emergency presentations at the CAP Tübingen from 2020 to 2024. The study was preregistered and approved by the ethics committee on 11/21/2018 (project number: 848/2018BO1).

**Results:**

We found that migrant adolescents tended to make less use of mental health services, with emergency presentations often serving as their first point of contact with mental health care. They were also more often diagnosed with posttraumatic stress disorder, anxiety disorder, or psychotic disorder. The role of a migration background in mental health crises remained unclear; however, financial strain and experiences of loss seemed to be prominent stressors, particularly in migrant adolescents.

**Conclusions:**

It remains clear that a migration background should be included as a risk factor for mental health crises rather than as an indicator. Migrant adolescents are exposed to heterogenous stress factors, thereby rendering it challenging to derive clear implications for treatment and emphasizing the need of further investigations. Yet, findings highlight the importance of facilitating access to lower threshold mental health services for migrant adolescents and the need for early identification of symptoms in order to prevent the escalation into acute emergencies.

**Supplementary Information:**

The online version contains supplementary material available at 10.1186/s12889-026-26964-7.

## Background

### Challenges in adolescence

Adolescence is a developmental period marked by multiple challenges in various life domains such as family, peers, school, and leisure [1, 2]. Stressors such as academic pressure, bullying, family instability, or interpersonal conflicts can negatively affect adolescents’ mental health and may lead to psychiatric disorders under certain circumstances [3, 4].

### Challenges in migrant adolescents

Children and adolescents with a migration background may face additional challenges, making them particularly vulnerable [5]. However, migration is a complex process, and each migrant’s experience involves unique circumstances and stressors, with coping responses varying considerably; thus, migrants cannot be considered a homogenous group [6, 7].

In this study, adolescents with a migration background are defined as individuals with a first-generation migration background (i.e., those not born in the host country) and individuals with a second-generation migration background (i.e., those born in the host country to parents who were born abroad) [8, 9]. The challenges they face vary according to generation status, socio-political conditions in the country of origin [9], and reasons for migration, including economic opportunities, forced migration due to political conflict, educational aspirations, or family reunification (cf. [6]). Individuals who migrate for economic opportunities typically report different pre-migration experiences than individuals who flee war, torture, or political persecution [6, 10]. In particular, refugees exposed to politically or religiously motivated violence often endure traumatic experiences prior to migration [6]. Furthermore, the stress associated with flight—frequently compounded by uncertainty regarding legal status in the host country—tends to be substantially higher than that experienced by individuals who migrate voluntarily [10]. Finally, post-migration adjustment is influenced not only by the reasons for migration but also by the sociopolitical context and reception conditions in the host country [6, 8].

Differences in stressors related to migration background also arise from the family’s cultural and religious context, and variations in stress burden have been observed across ethnic and religious groups [8]. The ability to maintain a cultural and religious identity is frequently considered a resilience factor among individuals with a migration background [6]. Migrants’ personalities and beliefs are also associated with adjustment to the host country’s culture, and cultural and religious identities often co-develop [6, 8]. On the other hand, adapting to the host country’s culture may be more challenging for migrants who have a high degree of pride for their own culture [6], and migrant adolescents also face additional challenges when navigating differing expectations between family and school [8, 11].

Thus, every migrant adolescent experiences unique stressors related to their migration background [6, 7]. These may include stressful experiences in the home country [6], acculturation stress [6], discrimination [12], and asymmetric acculturation within families [13]. Although migrant adolescents do not all share the same experiences, a migration background constitutes a risk factor for mental health problems [8].

### Mental illnesses in migrant adolescents

While migration itself is not a direct predictor of mental illness, it remains a significant risk factor, especially during adolescence, a critical period for identity development [8]. While some meta-analyses indicate a lack of clarity about whether migrants have higher rates of mental illness [8], it is evident that more than half of war refugees are suspected to suffer from mental disorders [10]. What is already assumed is that, in general, migrants, particularly first-generation migrants tend to experience higher rates of mental health problems compared with second-generation migrants [8, 9]. Yet, second-generation migrants may still face mental health issues, often linked to family dynamics related to migration [13, 14].

### Internalizing and externalizing mental illnesses

Migration is associated with both internalizing and externalizing mental health issues. However, there is no consistent association between a migration background and externalizing mental health issues [8]. The internalizing problems of migrant adolescents rated by their parents may be smaller than the problems of adolescents without a migration background rated by their parents because the parents of migrant adolescents may underestimate their children’s difficulties; this effect is explained by a fear of stigmatization or differing symptom awareness across different cultures [8]. Religious beliefs may also contribute to this effect [11, 15, 16], as mental illnesses—and suicide in particular—are regarded as religiously prohibited or morally impermissible in some traditions, such as Islam (cf. [15, 16]). Additionally, girls with a migration background are more likely to face externalizing problems than those without a migration background [8].

### Correlates of mental illnesses

Migrant families also often face socioeconomic challenges, which may contribute to issues such as loneliness, poor mental health, and lower life satisfaction [17]. Family dynamics, particularly mental health issues in parents, influence adolescent well-being [8]. Parenting dynamics, experiences of parental separation, and challenges related to cultural identity negotiation have also been identified as factors that contribute to mental health difficulties among migrant adolescents [8, 10]. However, family solidarity has proven to be a protective factor [18]. Poor integration and a lack of language skills are linked to mental health issues in adolescent migrants [6, 8]. Limited language proficiency is linked to lower academic performance [6, 8], however differences in the academic performances might also be explained by different standards towards academic success in different cultures. While many European schools and families in the host society emphasize autonomy and self-direction, value orientations differ across cultural contexts obedience and family cohesion [8]. Moreover, the process of adapting to the host country’s educational system may also affect academic performance [19].

Research indicates that adolescents with a migration background may experience unique challenges in peer relationships [8], although social support can serve as a resilience factor [6]. Stressful life events, harassment, and an external locus of control are also significant stressors for this population [6]. Discrimination is associated with internalizing problems in migrants [8] and often targets those who are most visibly different [20]. In the European Union, being Muslim appears to increase the risk of discrimination, particularly among those who display visible markers of their religion [21].

### Post-traumatic stress disorder

Many migrants, particularly first-generation migrants, experience elevated levels of anxiety, depression, or post-traumatic stress symptoms [9, 10]. One major factor may be the experience of traumatic events [10]. When individuals experience migration-related stressors, the nature and intensity of stress may differ depending on whether migration was voluntary or forced [6]. In particular, migration driven by political factors is associated with extremely high stress levels [6] and an increased prevalence of post-traumatic stress disorder (PTSD). Bhugra and colleagues [6] stated that migrants suffering from PTSD are also likely to experience more stressful events related to the process of migration (e.g., acculturation stress). Thus, it is also possible that migrants are more likely to be depressed because of traumatic events [6].

### Psychosis

Psychosis is also an area of concern, though findings remain mixed about whether psychosis results from migration or minority status [6]. Close and colleagues [9] explain this theory by stating that psychosis is often associated with discrimination, which is, in turn, linked to minority groups. In particular, discrimination most frequently targets those who are visibly identifiable as minorities [20]. Andleeb (2025) also found that the risk for psychosis among migrant adolescents is almost twice as high as among native peers, with Black and North African migrants being the most affected. Close and colleagues [9] also identified gender, ethnicity, and language barriers as factors that may increase risk, while emphasizing that migration alone does not automatically lead to psychosis. By contrast, according to the stress diatheses model explained in Bhugra and colleagues [6], psychosis may be a direct result of migration.

### Mental health crises in adolescents

Unmanaged stress with correspondingly high vulnerability may escalate into mental health crises with self-harming or suicidal behavior [22, 23] and can be followed by emergency presentations to child and adolescent psychiatric facilities. Increases in psychiatric emergencies have overwhelmed child and adolescent mental health services in recent years [24, 25].

### Connection between mental health crises and a migration background

Research has shown that migrant youths face more frequent emergency presentations [11, 26, 27] and a higher risk of suicidal thoughts [7, 8, 26, 28] compared with native youths around the world. The study by Lindert and colleagues [7] also suggested that second-generation migrants may be at even greater risk than their first-generation migrant parent. Even though studies on suicidal behavior and emergency admissions in migrant youth are limited, some of them have suggested that the risk varies by ethnicity [7, 11, 15, 28]. Others (e.g., [26]) postulated that refugees in general are at increased risk for major depression and accompanying suicidal thoughts, regardless of origin. Nevertheless, a migration background might not be the main factor for mental health crises leading to emergency admissions according to a study by Van der Post and colleagues [28], who could not find the effect after controlling for demographics and clinical characteristics. Additionally, migrants are also at a greater risk that emergency presentations will be their first contact with mental health care [27, 28].

Additionally, the relationship between suicidality and migration status seems to vary by the country of settlement [15]. Family dynamics [26], generational conflicts, and time spent in the host country [15] also seem to be linked to suicidality in migrant youth. Bhugra and colleagues [6] identified several risk factors for suicidality in migrant adolescents, including being female and residing in a rural area. Moreover, SES is associated with an increased use of emergency presentations and living in a deprived neighborhood, and a rural region is associated with the emergency presentation being the first contact with mental health care [27]. SES is also associated with suicidality in boys [6].

### Importance of research on mental health crises in adolescents with a migration background

Europe has experienced substantial migration flows in recent decades, shaping the demographic composition of many countries. Germany has seen a rise in migration in recent years, particularly from countries such as Syria, Ukraine, and Afghanistan [10, 29]. Nowadays, Germany´s migrants are mostly from Eastern and Central Europe [7]. As a result, nearly one third of all children and adolescents in Germany come from families with a migration background, and a substantial proportion are first-generation migrants [30].

### Provision and utilization of mental health care in migrant adolescents

There is a substantial proportion of adolescents with a migration background in Germany (e.g., [30]), and this group may encounter distinct challenges that can elevate their risk of mental health disorders including displacement, cultural adaptation, or experiences of discrimination (e.g., [26]). Despite this elevated risk, migrant adolescents tend to access psychiatric care less frequently than their native peers (e.g., [11, 28]), particularly outpatient services, but also inpatient or partial inpatient care (e.g., [16]).

This pattern is observed in other European countries as well. In Sweden, migrant adolescents utilize mental health services less than native adolescents, with utilization ratios ranging from 0.62 to 0.72 [31]. In the Netherlands, utilization ratios range from 0.63 to 0.91 [32]. Van der Post et al. (2011) further found that involvement in secondary mental health care, mainly outpatient services, was generally lower for migrants, although this was not true for all immigrant groups.

In contrast, some studies (e.g., [26]) found higher utilization of more intensive care, including inpatient psychiatric care for refugee minors and psychiatric emergency care for migrant adolescents [11, 16, 26, 27]. This combination of relatively low utilization of low-threshold mental health services and higher use of intensive care suggests substantial access barriers for migrant adolescents and indicates that treatment is often initiated at a late stage. Access is further restricted for asylum seekers, as treatment is typically provided only in acute emergencies [29].

### Previous findings on mental health crises of adolescent migrants

Whereas some (e.g., [33, 34]) meta-analyses have linked migration and mental health, there is not much data on stressors in adolescent clinical emergency populations. Since migrant children and adolescents are considered to be especially vulnerable, and adolescence often marks the beginning of mental disorders [35], it is important to understand migrant adolescents’ mental health. Due to the high risk for suicides and persistent damage to adolescents’ health (e.g., [36, 37]), it is necessary to take a closer look at the backgrounds of migrant adolescents in mental health crises. Only a few studies have specifically addressed acute mental health crises among adolescents with a migration background (e.g., [15]), and the results have been very heterogeneous.

This exploratory research therefore aims to examine mental health crises in migrant adolescents and compare stress factors between migrant and non-migrant adolescents with emergency presentations at the Child and Adolescent Psychiatry (CAP) Tübingen, with the goal of developing more targeted interventions and prevention strategies for migrant youth and to facilitate access to mental health care.

### Derivation of research questions

To ensure appropriate and accessible mental health support for migrant adolescents to help them avoid mental health crises, it is essential to first identify and understand the distinct stressors experienced by adolescents with and without a migration background. This knowledge can inform the development of culturally and contextually responsive interventions tailored to the specific needs of migrant youth.

First, we computed descriptive statistics on the sample characteristics of children and adolescents with and without a migration background who presented to the emergency department. Next, we applied exploratory inferential statistics to analyze the psychosocial burden between adolescents with emergency presentations with and without a migration background in order to determine which stress factors migrant adolescents with mental health crises are particularly likely to experience. Finally, we explored the differences within the sample of migrant adolescents in order to ultimately derive concrete treatment steps and preventive measures. Another objective was to gain more clarity on the previously heterogenous research findings. The study was based on data from patients with emergency presentations at the CAP Tübingen from 2020 to 2024.

#### Block A: Descriptive analyses of emergency presentations among adolescents with and without a migration background

- *Research Question (RQ) 1*: Compared to the general population in the region, the proportion of adolescents with a migration background with emergency presentations (outpatient emergency presentations and emergency admissions) is higher than the proportion of adolescents without a migration background.
- *RQ2*: Compared to the general population in the region, the proportion of adolescents with a migration background with emergency admissions is higher than the proportion of adolescents without a migration background.
- *RQ3*: Among adolescents with emergency presentations, the proportion of first contacts as emergency presentations is higher in those with a migration background than in those without a migration background.
- *RQ4*: Among adolescents with emergency presentations, the proportion of diagnoses of PTSD, anxiety disorders, and psychotic disorders is higher in those with a migration background than in those without a migration background.

#### Block B: Exploratory inferential analyses of psychosocial burden among adolescents with and without a migration background

- *RQ1*: Adolescents with emergency presentations who have a migration background do not differ from those without a migration background in terms of suicidality, risk to others, mental health problems (patient and parent perspectives), externalizing problems (patient and parent perspectives), and internalizing problems (patient perspective), when controlling for age, gender, socioeconomic status, place of residence, and ethnic background.
- *RQ2*: Adolescents with emergency presentations with a migration background show significantly higher internalizing problems from the parents’ perspective compared with those without a migration background, when controlling for age, gender, socioeconomic status, place of residence, and ethnic background.
- *RQ3*: Adolescents with emergency presentations with a migration background differ significantly from those without a migration background in:
  - ∙ Relationship quality (poorer family climate and more peer-related difficulties)
  - ∙ Socioeconomic status (lower SES)
  - ∙ Academic performance (lower school achievement)
  - ∙ Number of stressful life events (more frequent)
  - ∙ Parental burden (higher)

These significant differences hold when controlling for age, gender, place of residence, and ethnic background.

- *RQ3a*: Family climate explains a considerable proportion of variance in migration background (McFadden’s R² > 0.2).
- *RQ3b*: Descriptively, adolescents with emergency presentations and a migration background report experiencing disasters, bereavement, and financial hardship as stressful life events more frequently than those without a migration background.

#### Block C: Exploratory inferential analyses within the sample of migrant adolescents

Previous research has suggested that children and adolescents with a migration background are exposed to distinct experiences and contextual challenges that may influence their mental health. Consequently, variations in stress levels among adolescents facing high psychosocial strain can be expected. We aimed to conduct an exploratory investigation of differences in stress and suicidality within the sample of migrant adolescents.

- *RQ1*: Lower SES is associated with higher suicidality in migrant boys with emergency presentations but not in migrant girls with emergency presentations.
- *RQ2*: There is an association between migrant adolescents with emergency presentations living in rural areas and an increased likelihood of suicidal tendencies compared to those living in urban areas.
- *RQ3*: There is an association between migrant girls with emergency presentations and a higher likelihood of suicidal tendencies compared to migrant boys with emergency presentations.
- *RQ4*: There is an association between migrant adolescents with emergency presentations and a higher likelihood of suicidal tendencies when their parents have separated, compared to those whose parents have not separated.
- *RQ5*: Among migrant adolescents with emergency presentations, different ethnic groups differ in their associations for overall burden, internalizing and externalizing problems, suicidality, and family climate.

## Methods

The study was preregistered on the Open Science Framework [38], adhered to the Declaration of Helsinki and was approved by the ethics committee on 11/21/2018 (project number: 848/2018BO1). The aim of the study is to derive appropriate and accessible mental health support for migrant adolescents. We conducted an observational study with a two-group between-subjects design for which we collected data from patients at the CAP Tübingen. Data collection began in 2020 and is ongoing. There was no blinding in the study.

### Sample

We examined children and adolescents who presented to the CAP at the University Hospital Tübingen for emergency care, either through outpatient emergency consultations or emergency admissions. Informed consent was sought from all families including the patient and both legal guardians, who were typically the parents. Following the recommendation and approval of an amendment to the study in April 2024, data from families who provided data as part of the routine data collection in the CAP Tübingen were included in the analysis, even if they had not actively provided consent. The amendment acknowledged that the repeated inquiries for consent and the resulting strain, might function as an additional burden for families. Additionally the sample might be more representative encompassing a larger and more heterogeneous group of families. Data from families who actively declined participation were excluded.

The exclusion age was 18 years and older. Patients for whom insufficient data were available (missing questionnaire packages) were excluded from the analyses. Patients whose questionnaires were completed more than three months after the emergency presentation were also excluded from the analyses. If we could not determine whether a patient had a migration background, they were also excluded from the analyses.

Participants were categorized on the basis of the presence or absence of a migration background. There was no random assignment of patients to one group. Each individual was included in the analyses only once. In cases where both an outpatient emergency presentation and an emergency admission were documented for the same patient, only the more severe presentation – defined as the emergency admission – was included in the analyses. If multiple presentations of the same type were available, the case with the most comprehensive data was selected for inclusion. *N* = 194 (mean age: 14.7; 76.4% female; see Table 1) patients from the CAP Tübingen were included in the analyses, of which 67% were emergency admissions (see Table 2). For Research Question Blocks B and C, only 182 patients were analyzed due to missing questionnaires.

**Table 1:** Descriptive statistics for control variables

	Overall			No mb		mb		1st gen. mb		2nd gen. mb	
	NA	n	%	n	%	n	%	n	%	n	%
Migration backround	-	182	100	120	65.9	62	34.1	11	6.0	51	28.0
Sex^a^(female)	-	139	76.4	93	77.5	46	74.2	8	72.7	38	74.5
Parental separation (separated)	5	50	25.7	29	22.8	21	31.3	4	36.4	17	33.3
Place of residence (urban)	-	104	53.6	61	48.0	43	64.2	5	45.5	38	67.9
	NA M(SD)			M (SD)		M (SD)		M (SD)		M (SD)	
Age	- 14.69 (1.87)			14.58 (1.85)		14.88 (1.89)		14.87 (2.23)		14.88 (1.85)	

*N* = 182, *Mb* = migration background, *NA* = number of missing values, *n* = number of patients per group, *%* = percentage of the group (rounded) with the given characteristic, *M(SD)* = mean with standard deviation. *a* = one diverse

**Table 2:** Descriptive statistics for clinical characteristics

	Overall			No mb		mb		1st gen. mb		2nd gen. mb	
	*NA*	*n*	*%*	*n*	*%*	*n*	*%*	*n*	*%*	*n*	*%*
Migration background	-	194	100	127	65.5	67	34.5	11	5.7	56	28.9
Type of emergency presentation (admission)	-	130	67.0	87	68.5	43	64.2	5	45.5	38	67.9
Emergency presentation as first contact	8	96	49.5	60	47.2	36	53.7	6	54.5	30	53.5
Selected diagnoses	-										
Anxiety disorders		17	8.8	9	7.1	8	11.9	1	9.1	7	12.5
Schizophrenia, schizotypal, and delusional disorders		3	1.5	0	0	3	4.5	0	0	3	5.4
Reactions to stress and adjustment disorders		19	9.8	10	7.9	9	13.4	3	27.3	6	10.7
PTSD		3	1.5	1	0.8	2	3.0	1	9.1	1	1.8

*N* = 194, *Mb* = migration background, *NA* = number of missing values, *n* = number of patients per group. *%* = percentage of the group (rounded) with the given characteristic, *PTSD* = Post-Traumatic Stress Disorder

### Measured variables

No variables were manipulated. The group variable migration background (no migration background vs. migration background first and second generation) was assessed through citizenship and country of birth. Those who had a non-German citizenship or were not born in Germany were categorized as having a first-generation migration background. Those who had German citizenship and were born in Germany but at least one of their parents had only a non-German citizenship or was not born in Germany were categorized as having a second-generation migration background (developed by our department; see Additional File 1).

### Control variables

#### Socioeconomic status (SES)

The operationalization of socioeconomic status was based on the KiGGS Study Wave 1 (Children and Adolescents Health Survey Wave 1; [39]) and provided by parents. The SES calculation included the parents´ highest educational and professional qualifications and occupational status as indicators of education and employment. The CASMIN classification system (Comparative Analyses of Social Mobility in Industrial Nations; [40]) was used to classify educational levels. Occupational status was classified using the ISEI index (International Socio-Economic Index of Occupational Status; [41]), which refers to the ISCO-88COM classification [42]. Education and occupational status were assessed for each parent of the patient and any new partners of the parents. Each recorded person was assigned between 1 and 7 points for education and occupation, with decimal values for intermediate levels, allowing a maximum SES score of 14 points per person. In accordance with KiGGS [39, 43], the patient was assigned the highest SES of the parents living in the household, including possible partners. If data were missing for one recorded person, the highest SES of the remaining person(s) was assigned.

#### Ethnic background

Information about the birthplace of patients and the birthplaces and citizenships of parents were provided by parents (developed by our department; see Additional File 1). Categories from the Federal Statistical Office of Germany (Statistisches Bundesamt; [30]) were used to code the categories. Categories with less than 5% of families sharing the same migration background were combined with other categories.

#### Place of residence

Place of residence was derived from clinical files and categorized into urban (> 20,000 inhabitants) and rural (≤ 20,000 inhabitants).

#### Parental separation

Information about parental separation was provided by parents and coded into a binary variable (developed by our department; see Additional File 1).

### Clinical data

Information on the type of presentation was provided by the treating clinicians and coded into a binary variable. Information on previous treatment was queried as part of the routine care (non-validated items) and was therefore assessed by parents providing information on previous mental health care for different treatments (see Additional File 1). If information from parents about a previous treatment was provided, it was coded as previous contact with mental health care; otherwise, the emergency presentation was coded as first contact with mental health care. Diagnoses were derived from clinical files and coded in accordance with ICD-10 [44].

### Target variables

#### Suicidality and risk to others

Suicidality and risk to others were rated by responsible clinicians on a scale ranging from 1 (low) to 10 (high; developed by our department; see Additional File 1).

#### Overall burden, internalizing and externalizing problems

Overall burden was assessed with standardized questionnaires with a sum score of 112 items (excluding Item 113), each rated on a 3-point Likert scale from the Youth Self-Report (YSR; [45]), which captured the patients’ perspective. To capture the parents’ perspective, an additional sum score was derived from 112 items (excluding Item 113) of the Child Behavior Checklist (CBCL; [45]). Internalizing and externalizing problems were assessed with a sum score comprising 31 items that measured internalizing problems and 33 items that measured externalizing problems from the same questionnaires.

#### School performance

School performance was assessed with parents’ ratings of the question “How do you assess your child’s current performance in school?” on a 3-point Likert scale (below average, average, or above average; developed by our department as part of the routine care, not validated; see Additional File 1).

#### Parental burden

Parental burden was assessed from the parents’ perspective using a standardized questionnaire, the Brief Symptom Checklist (BSCL; [46]). A sum score was calculated from 53 items, each rated on a 5-point Likert scale.

#### Difficulties with friends

Difficulties with friends were assessed from the patients’ perspective using a standardized questionnaire, the friends scale of the KIDSCREEN-27 [47]. A sum score was calculated from 4 items, each rated on a 5-point Likert scale.

#### Family climate

Family climate was assessed from the patients’ perspective using a standardized questionnaire, the ROTH Familienklimaskalen [48]. A sum score was derived from 19 items, each rated on a 5-point Likert scale. The scale included three subscales: positive-emotional climate, organization, and control.

#### Stressful life events

Stressful life events were assessed with the Züricher Lebensereignisliste (ZLEL; [49]), a standardized questionnaire, by patients. The ZLEL contains 37 items covering school, family, friendships, and illness/accident/loss. Items that were given negative ratings (unpleasant or very unpleasant) were coded as stressful life events, whereas all others were coded as not stressful life events. A total sum score of stressful life events was calculated for each patient.

### Processing missing data

Patients who (1) were missing questionnaire packages, (2) filled out the questionnaires later than three months after the presentation, or (3) were missing information on migration background were excluded from the analyses. For usable questionnaires that were missing some data, the missing data were estimated with multiple imputation (number of datasets = 20, number of iterations = 10) in R. We used a machine learning approach (CART; [50]) to estimate the missing values.

### Analyses

Analyses were run in R Studio 2025.05.1 with R version 4.2.3. Besides descriptive analyses for the Block A research questions (only descriptively) and for Block B RQ3b, exploratory inferential analyses were calculated with an alpha level of 0.05. For Block B RQ1-RQ3a, multiple models were run: first, without adjusting for the covariates; second, with the covariates; and third, with interaction terms if applicable. For Block B RQ1 TOST-Tests [51] with covariates were calculated with an equivalence bound of Cohen´s d = ± 0.3. Equivalence bounds define the range within which differences between groups are considered negligible; if the observed difference lies within these bounds, the groups can be considered equivalent. For RQ2 and RQ3, linear regression models were calculated. For RQ3a, we computed a logistic regression and calculated McFadden’s R². For Block C, we computed linear regressions. None of the analyses were adjusted for multiple testing due to the exploratory nature of the study.

## Results

### Characteristics of emergency presentations of adolescent migrants (research questions in block A)

The first block of research questions addressed the sample characteristics of children adolescents with and without a migration background with emergency presentations descriptively. The sample consisted of 194 children and adolescents with emergency presentations at the CAP Tübingen. They had a mean age of 14.7 years (*SD* = 1.87), and 76% were female (Table 1). Mean age and gender proportions did not differ between the groups (*p* > .05). 34.5% of the children and adolescents were found to have a migration background, out of whom about 16.4% were considered first-generation migrants (Table 2). Compared with the population of migrant youth in the city of Tübingen (40%; [48]) and the federal state Baden-Württemberg (44%; [52]) descriptively, the proportion of children and adolescent migrants with emergency presentations at the CAP (34.5%) was lower than expected (Table 2). Additionally, the data did not support our descriptive RQ that the proportion of youth with emergency admissions and a migration background (64.2%; Table 2) would be higher than the proportion of those without a migration background (68.5%, Table 2). Most children and adolescents in the sample had a (South)eastern European migration background (15.4%), followed by a West and Central European migration background (4.4%) and a Middle Eastern Migration background (3.3%; Table 3). Out of the small number born in a country other than Germany, most were from (South)eastern Europe (3.1%), followed by Southern Europe (1.9%; Table 3). As expected, descriptive analyses showed that migrant adolescents were less likely to have had any contact with mental health care before their emergency admission. For 53.7% of adolescents (Table 2), the emergency presentation was their first contact with mental health care compared with 47.2% (Table 2) of non-migrant adolescents. As we expected, adolescent migrants with emergency presentations were more often diagnosed with anxiety disorders (11.9%); schizophrenia, schizotypal, and delusional disorders (4.5%); and PTSD (3.0%) than adolescents without a migration background (Table 2). Notably, schizophrenia, schizotypal, and delusional disorders were found only in second-generation migrants, whereas PTSD was more often found in first-generation migrants in relative terms (Table 2).

**Table 3:** Distribution of ethnic backgrounds

Ethnic background	Country of birth (1st gen. mb)^1^	Mb (1st and 2nd gen. mb)^2^
Germany	151 (94.2%)	122 (67.0%)
West- and Central Europe (BEL, AUT, CHE, LUX)	1 (0.6%)	8 (4.4%)
Southern Europe / Mediterranean region (ITA, GRC, PRT)	3 (1.9%)	5 (2.7%)
(South)eastern Europe / Balkans (HRV, SRB, BIH, SVN, SVK, POL, CZE, HUN, ROU, BLR, RUS)	5 (3.1%)	28 (15.4%)
Post-Soviet space / Central Asia (KAZ, KGZ, UZB)	0	5 (2.7%)
Middle East (TUR, LBN)	1 (0.6%)	6 (3.3%)
Africa (NGA)	0	1 (0.5%)
North America (USA)	1 (0.6%)	4 (2.2%)
South America (MEX, PER, BRA)	0	2 (1.1%)
British Isles (GBR, IRL)	0	1 (0.5%)

*N* = 182, *Mb* = migration background

^1^ 20 missing values

^2^ including children with one parent with a migration background, Country abbreviations found here: [53]

### Migration background as a correlate of mental health crises (research questions in block B)

Since the whole population of adolescents with emergency presentations is highly burdened, we expected that there would be no notable difference between the overall psychological burden between adolescents with and without a migration background. Therefore, we performed TOST-equivalence tests [51] to test for whether an effect between the two groups was absent for suicidality, risk to others, overall burden, internalizing problems (only from the patient perspective), and externalizing problems. On the basis of the equivalence bounds of *d* = -0.3 to 0.3, we did not find any significant effects (Tables 4 and 5) in the models without covariates or in the models with covariates, suggesting that equivalence could not be established. Additionally, the Hedges’ g values ranged from *g* ≈ 0.0 for suicidality and risk to others, *g* ≈ 0.1 for overall burden and externalizing problems (parents’ perception) and internalizing problems (patients’ perception), and *g* ≈ 0.2 for overall burden and externalizing problems (patients’ perception) in the models with covariates, indicating very small effect sizes and thus little differences between the groups. Confidence intervals (90%) for Hedges‘ g for overall burden, externalizing problems, and internalizing problems did include ± 0.3 bounds, further supporting the conclusion that the groups are not equivalent (Table 5). In an exploratory manner, we additionally ran null hypothesis tests (t-tests) to determine whether there was a difference between the two groups, which were also nonsignificant at *α* = 0.05 (Table 4). Therefore, the findings can be described as inconclusive, even though the difference between the groups seems negligible.

**Table 4:** Descriptive statistics for patient characteristics and results of equivalence analyses and tests of differences in target variables

	Descriptive statistics							Analyses			
	NA	M (SD)_overall_	M (SD)_no mb_	M (SD)_mb_	M (SD)_1st gen, mb_		M (SD)_2nd gen. mb_	*p*_TOST_	*p*_TOST−cov_	*p*_diff_	*p*_diff−cov_
Suicidality	3	5.06 (2.64)	4.96 (2.63)	5.26 (2.66)	3.18 (2.86)		5.71 (2.42)	0.502	0.412	-	-
Risk to others	3	1.64 (1.27)	1.69 (1.40)	1.54 (0.97)	1.55 (0.82)		1.54 (1.00)	0.234	0.241	-	-
Overall burden *Patient perspective* *Parent perspective* ^a^	^c^	191.62 (52.33) 164.58 (34.55)	191.27 (54.03) 164.28 (35.51)	192.29 (49.29) 165.16 (32.90)	175.00 (66.69) 161.09 (22.38)		196.02 (44.66) 166.04 (34.86)	0.535 0.543	0.914 0.706	- -	- -
Internalizing problems *Patient perspective* *Parent perspective*	^c^	57.75 (18.35) 53.00 (13.29)	57.68 (19.17) 53.01 (13.70)	57.89 (16.79) 52.98 (12.58)	50.91 (21.70) 46.09 (8.32)		44.51 (11.82) 54.47 (12.90)	0.487 -	0.811 -	- 0.991	- 0.457
Externalizing problems *Patient perspective* *Parent perspective*	^c^	43.09 (13.42) 44.52 (12.01)	42.84 (13.96) 44.23 (12.20)	43.55 (12.40) 45.10 (11.71)	39.09 (14.62) 47.64 (11.43)		44.51 (11.82) 4.55 (11.81)	0.577 0.619	0.896 0.776	- -	- -
School performance	21	1.99 (0.62)	1.89 (0.62)	2.18 (0.58)	2.20 (0.76)		2.18 (0.54)	-	-	0.004*	0.504
Parental burden	^c^	76.43 (32.11)	75.98 (30.91)	77.29 (34.57)	57.64 (25.80)		81.53 (34.95)	-	-	0.796	0.939
Difficulties with friends^b^	^c^	12.04 (5.29)	11.47 (5.28)	13.16 (5.16)	15.27 (5.53)		12.71 (5.01)	-	-	0.040*	0.609
Stressful life events	^c^	7.13 (4.37)	7.39 (4.45)	6.61 (4.20)	7.09 (3.99)		6.51 (4.27)	-	-	0.257	0.580
Socioeconomic status	14	8.57 (1.64)	8.69 (1.59)	8.33 (1.74)	8.55 (1.51)		8.55 (1.51)	-	-	0.179	0.028*
Family climate	^c^	57.55 (19.25)	58.43 (18.11)	55.84 (21.33)	48.00 (29.75)		57.53 (19.01)	-	-	0.390	0.182

*N* = 182, *NA *= number of missing values before multiple imputation, *M(SD*) = mean with standard deviation, *mb* = migration background. *pTOST* = result of TOST equivalence test (max. *p*-value) with and without covariates (gender, sex, socioeconomic status, place of residence, and ethnic background) between the groups with and without mb, *pdiff* = result of linear regression on target variable between the groups with and without mb with and without covariates (gender, sex, socioeconomic status, place of residence, and ethnic background)

^a^ Item 60 was removed since estimation was not possible due to floor effects

^b^ higher score means less difficulties

^c^ Missing values for individual items were imputed, and a total score was computed

* significant at α < 0.05

**Table 5:** Results of equivalence testing

	TOST without covariates							TOST with covariates				
	NA		β	90%-CI β	p	Hedges’ g	90%-CI g	β	90%-CI β	p	Hedges’ g	90%-CI g
Suicidality		3	0.30	-0.38; 0.99	0.502	0.11	-0.14; 0.36	0.14	-1.05; 1.33	0.412	0.03	-0.22; 0.28
Risk to others		3	-0.15	-0.49; 0.18	0.234	-0.12	-0.37; 0.14	-0.06	-0.63; 9.52	0.241	-0.02	-0.28; 0.23
Overall burden Patient perspective Parent perspective^a^		^b^	1.02 0.89	-12.55; 14.59 -8.07; 9.85	0.535 0.543	0.02 0.02	-0.23; 0.27 -0.22; 0.27	-17.21 -5.55	-37.57; 3.15 -21.56; 10.46	0.914 0.706	-0.21 -0.09	-0.46; 0.04 -0.34; 0.16
Internalizing problems Patient perspective		^b^	0.20	-4.56; 4.96	0.487	0.01	-0.24; 0.26	-4.17	-11.40; 3.06	0.811	-0.14	-0.40; 0.12
Externalizing problems Patient perspective Parent perspective		^b^	0.71 0.87	-2.77; 4.19 -2.24; 3.99	0.577 0.619	0.05 0.07	-0.20; 0.30 -0.18; 0.32	-4.63 -2.81	-10.27; 1.02 -8.29; 2.66	0.896 0.776	-0.21 -0.13	-0.46; 0.05 -0.38; 0.12

*N* = 182, *TOST* = Two One-Sided Tests with equivalence bounds d = +/- 0.3, *NA* = number of missing values before multiple imputation *β* = unstandardized regression coefficient, *90%-CI β *= 90% confidence interval for *β*, *p* = result of TOST equivalence test (max. p-value), *Hedges’ g* = standardized mean difference, *90%-CI g* = 90% confidence interval for Hedges‘ g, *Covariates* = gender, sex, socioeconomic status, place of residence, and ethnic background

^a^ Item 60 was removed since estimation was not possible due to floor effects

^b^ Missing values for individual items were imputed, and a total score was computed

To test whether there were differences between parents´ views of internalizing problems in adolescents with and without a migration background, we computed linear regression models, which did not yield any significant results at α = 0.05, even after controlling for confounding covariates and including exploratory interactions between migration background and the covariates in the model (Table 4).

We also calculated linear regression models to test whether relationship quality differed between migrant adolescents and those without a migration background. For family climate, migration background was not a significant explanatory variable in any of our models (Table 4). However, age and gender seemed to play a role in differences in the family climate (both *p*s < 0.05). We conducted an exploratory test of whether the interaction between age and migration background and gender and migration background played a role in predicting the family climate but found that they did not (both *p*s > 0.05). Contrary to the findings for relationship quality with family, we found that a migration background seemed to play a role in peer relationships, but in the opposite direction than expected. In the model without covariates, difficulties with friends yielded a significant (*p* = .040) result such that migrant adolescents had fewer peer-related difficulties. However, this significant finding disappeared when we added the covariates (Table 4). Comparing models predicting difficulties with friends, the model with migration background as an explanatory variable was not significantly better (*p* > .05). We additionally tested for whether migration background explained a considerable proportion of the variance in family climate, which was not the case. The logistic regression model revealed a *McFadden’s R²* of < 0.01.

Migration background was found to be a significantly associated with SES in the model with covariates (*p* = .028) such that migrant adolescents had a lower SES, but the result was not significant when the other covariates were not included (Table 4). Comparing models, migration background was significantly associated with SES (*p* = .028). Ethnic background was not correlated with SES, nor was the interaction between migration background and ethnic background (*p* = .100). School performance was rated significantly better, by parents, for non-migrant adolescents in comparison with migrant adolescents, in the model without controlling for covariates (*p* = .004). When the covariates were included, migration background was not associated with school performance (Table 4). Yet SES and gender were significantly correlated with school performance.

Migrant adolescents reported a smaller number of stressful life events on average than non-migrant adolescents, a finding that was not expected (6.61 for migrants and 7.39 for non-migrants; Table 4). However, the regression results were not significant at *α* = 0.05. Nevertheless age, gender, and place of residence were significantly associated with the number of stressful life events (*p* < .05). While the interaction between migration background and age was not statistically significant (*p* = .054), it was close to the predefined significance threshold (*α* = 0.05). We separately examined the specific stressful life events *disasters*, *bereavement*, and *financial hardship* more closely on a descriptive level for the sample size of *n* = 182, as these events were expected to occur more frequently in migrant adolescents. Specifically, disasters were most often reported by non-migrant adolescents (2.4%) compared with no reports by first-generation migrant adolescents and 1.6% by second-generation migrant adolescents. To assess bereavement, we analyzed deaths and absence with the result that deaths were reported most frequently in first-generation migrant adolescents (18.2%), followed by adolescents without a migration background (15.4%) and second-generation migrant adolescents (11.5%) and, whereas absence was most often reported by adolescents without a migration background (9.8%) and were reported at a rate of only 3.2% by second-generation migrants. None were reported by first-generation migrants. Lastly, financial hardship was mostly reported as a stress factor by first-generation migrant adolescents (18.2%) and second-generation migrant adolescents (13.6%), while only 8.4% of non-migrant adolescents reported financial hardship.

Finally, parental burden was rated a little higher by parents of children with a migration background, especially for those with a second-generation migration background (Table 4). However, this observation was not statistically significant in any model (Table 4).

### Variations in stress factors associated with mental health crises in migrant adolescents (research questions in block C):

Variations in stress factors among adolescents facing mental health crises can be expected, which we analyzed on an exploratory level in this study. For the given sample size of *n* = 62 (*n* = 11 for first generation; *n* = 56 for second generation) adolescents with a migration background, the data did not support the expected association between lower SES and higher suicidality in migrant boys (*p* = .629). We also did not find that migrants in rural areas (*p* = .486) and migrant girls (*p* = .128) were more likely to be suicidal, nor did we find that migrants in rural areas had a greater overall burden (*p* = .839). However, migrant girls’ self-reports showed that they did have a greater burden (*p* = .008). The data did not support our expectation of more overall distress in migrant adolescents with separated parents (*p* = .762 for self-reports, *p* = .818 for parent reports, and *p* = .524 for suicidality by clinicians’ reports).

We also took a closer look at different ethnic groups associated with distress. When we entered the different ethnic groups (Table 3) as an explanatory variable, the regression results were not significantly associated with suicidality, overall burden, internal and external burden, or family climate (all *p*s > 0.05). Lastly, when we used more specific information about migration background (i.e., Balkan and Middle Eastern backgrounds) as explanatory variables, neither having a Balkan background nor having a Middle Eastern background was significantly associated with the stressors mentioned before (both *p*s > 0.05).

## Discussion

### Discussion of the findings

#### Block A: Descriptive analyses of emergency presentations among adolescents with and without a migration background

Unexpectedly, the number of migrant children and adolescents presenting as emergencies at the CAP was smaller in relation to the number of migrant children and adolescents in the general population. Since it can be assumed that adolescents with a migration background often experience additional stressors [5] and are more frequently affected by mental disorders [8], it is surprising that they did not present more often as emergencies at the CAP Tübingen. One possible explanation could be methodological in nature: The current sample might not adequately represent patients with a migration background, as many adolescents with limited German language skills were unable to complete the questionnaires and were therefore not included in the analysis. Nevertheless, during data preparation, efforts were made to exclude as few incomplete cases as possible by addressing missing data through multiple imputation. The use of the machine learning approach CART is both timely and reliable for estimating missing values [50]. Another explanation for rejecting the hypotheses of a larger proportion of migrant adolescents with emergency presentations (Block A, RQ1 & RQ2) may be that adolescents with a migration background, although often highly burdened, indeed present less frequently as emergencies at CAP services. Empirical findings suggest that some groups of individuals with a migration background have less knowledge about available services or face higher thresholds for seeking help from support systems [8, 9]. In addition to structural barriers, religious beliefs may also influence help-seeking behavior toward mental health care [15, 16]. For example, studies have shown that young Turkish women are more likely to commit suicide than their native counterparts [7, 54, 55]. This highlights the importance of accessible and culturally sensitive emergency care that may help identify and address suicidal crises at an earlier stage.

In the present study, emergency presentations at the CAP were more often the first point of contact for adolescents with a migration background compared with those without a migration background. This finding supports our RQ (Block A, RQ3) and aligns with previous literature [27, 28]. While this result requires cautious interpretation due to the use of a non-validated questionnaire (intended to minimize missing data and patient burden), it fits with the aforementioned thought that migrants may have limited knowledge about available mental health care services, have a higher threshold for seeking help [8, 9], or face stigma [8, 10, 16]. The underrepresentation of migrants in child and adolescent psychiatric services may also be related to structural or financial barriers that restrict access to lower threshold mental health care. For example, asylum seekers often face limited insurance coverage [28], and families with fewer socioeconomic resources may struggle to access private services, leading to longer waiting times [56]. These barriers, more common among some migrant populations (e.g., [17]) contribute to the relatively low utilization of lower threshold mental health care, as confirmed in our study. In contrast, the higher utilization of emergency mental health care, as indicated by previous research (e.g., [11, 16, 26, 27]), raises concerns that treatment for adolescents with a migration background is often initiated too late.

Concerning diagnoses at the time of the emergency presentations (Block A, RQ4), we expected PTSD, anxiety disorders, and psychotic disorders to be more frequent in migrant adolescents. In the present study, psychotic disorders were indeed more prevalent among adolescents with a migration background (see also [6, 9]). Interestingly, psychosis diagnoses were found exclusively in second-generation migration background patients. This finding is consistent with previous literature that describes the risk of the development of a psychotic disorder as a result from the stress of the process of migration and particularly as the adjustment for exposure to discrimination (e.g. [9]) or especially for second generation migrants the stress of adjusting in bi-cultural settings [6]. PTSD diagnoses were also more frequent among migrant adolescents (see also [9, 10]). It should be noted that the present sample predominantly comprises adolescents with a second-generation migration background, whereas only a small proportion are first-generation migrants. Since first-generation migrant adolescents are more likely to have experienced potentially traumatic events (cf. [10]), particularly in the context of politically motivated migration, the prevalence of PTSD might be even underestimated in the present study. However, migration does not necessarily imply exposure to traumatic experiences, and a differentiated assessment of migration-related experiences is therefore essential [6, 10]. In our sample, most adolescents with a migration background originated from Southern and Southeastern Europe. Future studies should assess specific reasons for migration in greater detail. However, based on the OECD statistical database, it can be assumed that a substantial proportion of these migrations may have been economically motivated [57]. Against this background, the finding that PTSD diagnoses were more frequent among migrant adolescents should be interpreted with caution, particularly given the small number of PTSD cases in the total sample. Anxiety disorders were likewise more prevalent among patients with a migration background, a finding that aligns with previous studies [9, 10] as well.

#### Block B: Exploratory inferential analyses of psychosocial burden among adolescents with and without a migration background

We expected that the overall burden would be high among all adolescents with emergency presentations at the CAP (Block B, RQ1). Yet neither the equivalence test showed equivalence between the groups when comparing those with and without a migration background, nor the explorative difference-based test turned out to be statistically significant; thus the result can be described as inconclusive. However, we assume a comparable burden in all examined adolescents due to the observed differences in means and confidence intervals for the effect size and suggest that a larger sample size may be necessary to achieve statistical equivalence [51]. Nevertheless, we expected the perceived burden of migrant adolescents rated by their parents to be smaller than for those without a migration background. Belhadj Kouider and colleagues [8] explained an effect such as this one by suggesting that, due to fear of stigmatization and differences in symptom awareness across cultures, parents of adolescents with a migration background may underestimate their children’s difficulties. However, contrary to our RQ (Block B, RQ2) and the previous literature [8], the perceived burden of adolescents with a migration background did not differ from that of adolescents without a migration background as rated by their parents.

Previous studies have shown that difficulties in families are often associated with family dynamics [8]. Parental relationship dynamics [8, 10] and issues related to cultural identity [8] are particularly likely to be associated in families with a migration background. Studies have suggested that certain stressors, which may be more prevalent in families with a migration background (e.g., acculturation processes, experiences of discrimination, or socioeconomic challenges; (see [8, 10]), can be associated with a higher risk of intrafamilial conflict, which may in turn affect the overall family climate.

In the present study, this trend was observed on a descriptive level, but it did not turn out to be substantial or statistically significant (Block B, RQ3 & RQ3a). One possible reason might be the rather small sample size. Moreover, the use of only some subscales of the short version of the family climate scale [48] should be reconsidered in future studies concerning similar research questions, since the intellectual-cultural orientation dimension was not captured in this study, although it appears to be highly relevant in this context. Beyond family climate, parental burden also plays a role in adolescent mental health (e.g., [8, 10]). However, no significant differences in parental burden were found between adolescents with and without a migration background, despite trends in the expected direction (Block B, RQ3), namely, that parents in families with a migration background experienced higher levels of stress (e.g., [10]). Regarding intrafamilial relationships, the findings on peer-related difficulties also could not be reconciled with previous literature [8]. The expectation that there would be an association between having a migration background and facing greater challenges in friendships compared with those without a migration background (Block B, RQ3) was not supported by the data. On the contrary, in the short questionnaire used in the present study, adolescents with a migration background even rated the quality of their friendships as higher. However, the significant effect disappeared once the covariates were added to the model. In the context of the European Union, perceived discrimination tends to be highest among Black migrants, migrants from (South) Africa [20], and Muslim individuals [21]. Yet the sample in this study primarily consists of adolescents with a migration background from European regions with predominantly Christian cultural traditions, who may generally experience more favorable social environments. Since these groups are better represented in the present sample, this may partly account for the relatively positive evaluations of social relationships reported by the adolescents.

Consistent with previous findings, SES was lower among adolescents with a migration background than among those without a migration background (e.g., [17]). Moreover, the hypothesis that facing greater academic performance challenges would be associated with a migration background (Block B, RQ3) was supported (see also [8, 10]). This study did not assess students’ actual performance directly, but parents were asked to evaluate their children’s achievement using a non-validated item as part of routine care. Interestingly, these parental assessments differed between adolescents with and without a migration background, raising the question of whether parents in families with a migration background may generally hold higher academic expectations (e.g., [8]). Both lower SES and poorer academic performance may be linked to a lack of language skills, which, according to Bhugra and colleagues [6] and Belhadj Kouider and colleagues [8], are themselves associated with mental health problems. In addition, academic performance may depend on the length of time spent in the host country’s educational system (cf. [19]) or on discrepancies between parental academic expectations and students’ actual grades (cf. [8]). Future studies should therefore assess adolescents’ language skills and objectively measured academic performance more comprehensively in order to disentangle these interrelated factors.

Unexpectedly, the number of reported stressful life events was descriptively lower among adolescents with a migration background compared with those without a migration background (Block B, RQ3). Even the average number of stressful life events among first-generation migrant adolescents was lower than that of adolescents without a migration background. This finding is particularly surprising, since some adolescents who have migrated themselves might experience extremely stressful events such as war, violence, poverty or forced migration (e.g., [9, 10]) as part of the migration process. However, the number of patients with a first-generation migration background in this sample was very small. None of the observed differences in the number of stressful life events turned out to be statistically significant, which suggests that there were no differences between adolescents in the number of stressful life events. This might be partly explained by the reason for migration, which is expected to be mostly of an economic nature for European migrants [57], who constitute the majority of the sample. Another possible explanation is that the interpretation of what is classified as a disaster differs between adolescents such that adolescents with a migration background may have become somewhat hardened by their past experiences and therefore perceive disasters less strongly as such. Even so, descriptively, migrant adolescents reported more instances of deaths and financial hardship than those without a migration background (Block B, RQ3b), which is in line with previous research (e.g., [9, 10]). However, they did not report experiences of disasters more often, which would have been expected (Block B, RQ3b), especially for first-generation migrant adolescents (cf. e.g., [9, 10]).

#### Block C: Exploratory inferential analyses within migrant adolescents

Since previous research has suggested that children and adolescents with a migration background are exposed to different challenges and are vulnerable to varying degrees (e.g., [6]), we expected variations in stress levels and suicidality among migrant youth facing high psychosocial strain. Based on previous literature, we assumed that certain factors would be associated with burden among migrant adolescents (Block C). However, the data did not support any of these assumed associations. According to Bhugra and colleagues [6], a lower SES is associated with higher suicidality in migrant boys presenting as emergencies compared with migrant girls. Belhadj Kouider and colleagues [8] argued that navigating between two different value systems — the host country’s and the family’s — is particularly challenging for boys. However, this effect was not found in the present study (Block C, RQ1). Similarly, the expectation that suicidality would be higher among migrant adolescents living in urban areas (Block C, RQ2; [6]) was not supported; yet results in other studies also have not detected associations between the degree of urbanization and mental illness (e.g., [9]). We also did not find support for the RQ that migrant girls would be more likely to be suicidal than migrant boys (Block C, RQ3; (see [6]), although migrant girls reported higher levels of overall burden in our sample. The theory of higher suicidality following parental separation among migrant adolescents (see [26]) also was not supported (Block C, RQ4). Notably, the data did not allow us to determine whether parental separation occurred before or after migration, which might have been of interest.

Additionally, larger ethnic subgroups (here, adolescents with a Balkan or Middle Eastern migration background) were examined separately with regard to burden and family climate (Block C; RQ5). The analyses revealed that ethnic groups were not correlated with either burden or family climate. Some earlier studies (see, e.g., [8]) have examined specific ethnic groups with respect to their burden and have found different results for different ethnic groups to some extent. Additionally, there seem to be differences between different religions, and there might be differences between ethnic groups, with a reinforcement of burden in non-Europeans. As previously noted, discrimination seems to be more prominent among Black or African migrants and those with a Muslim background [20, 21]. One possible reason for the lack of differences among migrant adolescents may therefore be the limited sample size, which may have resulted in insufficient statistical power to detect small effects, especially within individual ethnic subgroups.

Since each individual brings unique experiences and challenges, several studies have emphasized that migrant adolescents cannot be regarded as a homogeneous group (e.g., [6, 7]). Although all adolescents with a migration background may face a more difficult developmental process (e.g., [6]), these factors can affect psychological well-being in different ways and, depending on resilience factors, like social support or self-esteem (e.g., [6]), may lead to increased levels of strain. Hence, there is a need to determine whether it makes sense to divide adolescents with a migration background into specific groups or whether it is necessary to consider each adolescent on a purely individual basis. For now, it remains to be determined whether migrant adolescents nevertheless have specific risk factors that contribute to their mental health crises.

### Strengths and limitations

Due to the previously mentioned limitations, particularly the limited representativeness of children and adolescents with a migration background, the relatively small sample size, and the recruitment from a single clinical site, caution is warranted when generalizing the findings beyond this specific setting. The smaller sample size — which, according to GPower calculations, resulted in a power of 75% assuming that the effects observed are very small (0.03) — may also have reduced the likelihood of detecting subtle group differences. Given the exploratory nature of the analyses and the absence of adjustment for multiple testing, all results should be interpreted cautiously and primarily as hypothesis-generating. Nevertheless, the findings can still be interpreted in terms of correlational relationships and are valuable for future research on mental health crises in migrant adolescents.

The analysis of individual ethnic groups aligns with the most recent lines of research. From a methodological perspective, bias was minimized as much as possible through the multiple imputation of missing data, the inclusion of a wide range of covariates, and the use of almost exclusively validated measurement instruments, however a few instruments were not validated. This study provides new insights into the still very limited and heterogeneous body of research on mental health crises among adolescents with a migration background and raises further questions. The sample itself is very valuable, as it represents the most severely affected adolescents with mental disorders, and the study links mental health crises and challenges of migrant adolescents in order to find adequate interventions. To our knowledge, this study is the first of its kind conducted in Germany and provides important insights for the Central European context into the characteristics of adolescents with a migration background presenting to CAPs. However, differences in regional healthcare structures, referral pathways, and the composition of migrant populations across Central Europe [16, 58] substantially influence clinical profiles and help-seeking behavior, warranting caution when generalizing these findings beyond comparable emergency care settings. Nevertheless, the results inform future research and contribute to improving access to mental health services and the development of culturally sensitive treatment approaches for migrant adolescents.

### Implications and further research

Adolescents presenting to the CAP as emergencies represent one of the most severely burdened groups of young people. Timely and adequate intervention is thus indispensable for preventing profound long-term implications and the substantial disruption of developmental trajectories. At the same time, a migration background may entail specific developmental challenges and additional stressors. To date, these two aspects have rarely been examined in conjunction, leaving a considerable gap in research and practice. The present study contributes to the still highly heterogeneous body of evidence regarding the role of migration background in adolescents experiencing mental health crises and raises new questions for future research. Despite the limitations mentioned above, several implications can be derived.

First, it appears that no uniform treatment scheme can be developed at this point, as migrant adolescents present with a wide range of difficulties. It must be emphasized that a migration background should not be regarded as a direct predictor of psychological crises but rather as one potential risk factor that interacts with multiple other determinants. Nevertheless, several patterns emerged. Migrant girls with emergency presentations appear to be more burdened overall than migrant boys with emergency presentations. Migrant adolescents with emergency presentations may have stronger peer relationships, yet they tend to come from lower socioeconomic backgrounds and are more often rated by their parents as performing poorly in school. Deaths and financial hardship were reported more frequently, particularly among first-generation migrants. By contrast, fewer adolescents with a migration background appeared as emergencies at the CAP compared with adolescents without a migration background. Considering the higher burden, this discrepancy is striking and raises the question of what happens to those who do not present at CAPs, whether they are at increased risk of suicide or rely on alternative coping mechanisms. In addition, adolescents with a migration background with emergency presentations reported fewer prior contacts with mental health services, thereby raising the concern that many seek help too late. Anxiety, psychosis, and PTSD were more frequent in migrant adolescents than in adolescents without a migration background presenting as emergencies, thereby suggesting that migrant adolescents may be more vulnerable to these mental disorders and highlighting the need to identify and treat symptoms at an earlier stage.

These findings highlight the urgent need for further studies with larger sample sizes and strategies to ensure more representative inclusion of adolescents with a migration background within the group of CAP emergencies. Future research should especially investigate why adolescents with a migration background are less likely to appear in CAP emergency services and how access to mental health care can be facilitated. At present, the emphasis should be on improving accessibility to services rather than on tailoring highly specific interventions, while at the same time considering early screening for certain diagnoses.

## Conclusion

As previous research shows, migrant adolescents are at increased risk for mental health disorders and represent a considerable proportion of the adolescent population worldwide, including in Germany. Thus, it is crucial to ensure accessible and appropriate mental health care in order to prevent mental health crises in adolescent migrants. As of now, there is a considerable gap in research on migrant youth mental health crises. Identifying specific stressors among migrant adolescents is therefore essential. However, the few current findings on mental health crises in this group remain heterogeneous, highlighting the need for further research. One of the main findings of this study is that migrant adolescents are more likely to seek emergency services as their first encounter with mental health care, indicating that they tend to underutilize other mental health services. This underscores the importance of facilitating access to care. Also, migrant adolescents are more frequently diagnosed with PTSD, anxiety disorders, and psychotic disorders, highlighting the need for early identification of symptoms in order to prevent the escalation into acute emergencies. Yet the role of a migration background in mental health crises remains insufficiently understood, as current findings are somewhat inconsistent with previous research. Nevertheless, financial strain and experiences of loss appear to be particularly prominent stressors among migrant adolescents. At the same time, they are exposed to heterogeneous stress factors, which makes it difficult at present to derive clear implications for treatment. Overall, a migration background should not be considered a direct predictor of mental health crises but instead as a potential risk factor especially concerning the utilization of mental health services. While the results are embedded in a specific national context, the mechanisms related to crisis development and patterns of service utilization may extend beyond this setting and warrant examination in other countries. Further investigation is therefore urgently needed.

## Supplementary Information

Supplementary Material 1.

## Data Availability

The datasets analyzed during the current study are not publicly available due to limited consents from the families but may be available from the corresponding author on reasonable request.

## Abbreviations

CAP: Child- and Adolescent Psychiatry
PTSD: Post-Traumatic Stress Disorder
RQ: Research Question
