# Supplementary material for: Mental health crises among youth in the context of migration – a cross-sectional study of psychiatric emergencies
Source: BMC Public Health. 2026 Mar 19;26:1164. doi: 10.1186/s12889-026-26964-7 (PMC13063658; doi:10.1186/s12889-026-26964-7)
Supplement: Supplementary file 1 — Supplementary Material 1. [file 12889_2026_26964_MOESM1_ESM.docx]

# Additional File 1 – Translation of questionnaires used in the study

## Original Version in German

### Excerpt from the questionnaire for parents for anamnesis assessment

Used to assess migration and ethnic background, parental separation, previous treatment and school performance

**Kind/Jugendlicher**

*Geburtsort: [open text]*

*Kind lebt bei… [open text]*

1. Leiblichen Eltern [Checkbox for 'Yes']
2. Leiblicher Mutter [Checkbox for 'Yes']
3. Leiblichem Vater [Checkbox for 'Yes']
4. Pflegeeltern [Checkbox for 'Yes']
5. Wohngruppe [Checkbox for 'Yes']
6. Adoptiveltern [Checkbox for 'Yes']

**Mutter**

*Nationalität: [open text]*

*Geburtsort: [open text]*

**Vater**

*Nationalität: [open text]*

*Geburtsort: [open text]*

**Lebenssituation der Eltern**

*a) getrennt [Checkbox for 'Yes']*

*b) geschieden [Checkbox for 'Yes']*

**Bisherige Behandlungen/Therapien**

*Was haben Sie oder andere (z. B. Schule, etc.) bereits versucht, um die Problematik zu verbessern? [open text]*

*Bisherige Behandlungen/Therapien [Details in age, duration, and additional description where applicable]*

a) Lerntherapie

b) Psychologische Beratungsstelle

c) Ambulante Psychotherapie

d) Kinderpsychiatrische Behandlung

e) Teilstationäre Psychotherapie (Tagesklinik)

f) Stationäre Psychotherapie

g) Ambulante Krisenintervention/Notfallvorstellung

h) Stationäre Krisenintervention

i) Medikation

**Schule**

*Wie beurteilen Sie die gegenwärtige Leistung Ihres Kindes in der Schule? [Check the most approriate box]*

1. überdurchschnittlich
2. durchschnittlich
3. unterdurchschnittlich

### Excerpt from the questionnaire for responsible clinicians at the time of presentation

Used to assess type of presentation, suicidality and risk to others

**Art der Vorstellung** [Check the approriate box]

1. Ambulante Krisenvorstellung
2. Stationäre Aufnahme

**Einschätzung**

Suizidalität [Check the most approriate box]

1. 1
2. 2
3. 3
4. 4
5. 5
6. 6
7. 7
8. 8
9. 9
10. 10

Fremdgefährdung [Check the most approriate box]

1. 1
2. 2
3. 3
4. 4
5. 5
6. 6
7. 7
8. 8
9. 9
10. 10

## Translated into English

### Excerpt from the questionnaire for parents for anamnesis assessment

Used to assess migration and ethnic background, parental separation, previous treatment and school performance

**Child/Adolescent**

*Place of birth: [open text]*

*Child’s living arrangement*

Child lives with … [open text]

1. Biological parents [Checkbox for 'Yes']
2. Biological mother [Checkbox for 'Yes']
3. Biological father [Checkbox for 'Yes']
4. Foster parents [Checkbox for 'Yes']
5. Residential group [Checkbox for 'Yes']
6. Adoptive parents [Checkbox for 'Yes']

**Mother**

*Nationality: [open text]*

*Country of birth: [open text]*

**Father**

*Nationality: [open text]*

*Country of birth: [open text]*

**Parents' living situation**

*a) separated [Checkbox for 'Yes']*

*b) divorced [Checkbox for 'Yes']*

**Previous treatment**

*What steps have you or others (such as the school) already taken to address the issue? [open text]*

*Previous Treatments/Therapies [Details in age, duration, and additional description where applicable]*

a) Learning therapy

b) Psychological counseling center

c) Outpatient psychotherapy

d) Child psychiatric treatment

e) Partial hospitalization

f) Inpatient psychotherapy

g) Outpatient crisis intervention/emergency presentation

h) Inpatient crisis intervention

i) Medication

**School**

*How do you assess your child’s current performance in school? [Check the most approriate box]*

1. above average
2. average
3. below average

### Excerpt from the questionnaire for responsible clinicians at the time of presentation

Used to assess type of presentation, suicidality and risk to others

**Type of presentation** [Check the approriate box]

1. Outpatient emergency presentation
2. Emergency admission

**Assessment**

Suicidality [Check the most approriate box]

1. 1
2. 2
3. 3
4. 4
5. 5
6. 6
7. 7
8. 8
9. 9
10. 10

Risk to others [Check the most approriate box]

1. 1
2. 2
3. 3
4. 4
5. 5
6. 6
7. 7
8. 8
9. 9
10. 10
